# Survey of Insomnia and Related Social Psychological Factors Among Medical Staff Involved in the 2019 Novel Coronavirus Disease Outbreak

**DOI:** 10.3389/fpsyt.2020.00306

**Published:** 2020-04-14

**Authors:** Chenxi Zhang, Lulu Yang, Shuai Liu, Simeng Ma, Ying Wang, Zhongxiang Cai, Hui Du, Ruiting Li, Lijun Kang, Meilei Su, Jihui Zhang, Zhongchun Liu, Bin Zhang

**Affiliations:** ^1^Department of Psychiatry, Nanfang Hospital, Southern Medical University, Guangzhou, China; ^2^Guangdong-Hong Kong-Macao Greater Bay Area Center for Brain Science and Brain-Inspired Intelligence, Guangzhou, China; ^3^Department of Psychiatry, RenMin Hospital of Wuhan University, Wuhan, China; ^4^Department of Psychiatry, Jing Men No. 2 People's Hospital, Jingmen, China; ^5^Department of Psychiatry, The Chinese University of Hong Kong, Hong Kong, China

**Keywords:** insomnia, medical staff, COVID-19, mental health, sleep quality, stress, isolation, outbreak

## Abstract

**Objective:**

The outbreak of the 2019 novel coronavirus disease (COVID-19) not only caused particularly large public health problems, but also caused great psychological distress, especially for medical staff. We aimed to investigate the prevalence rate of insomnia and to confirm the related social psychological factors among medical staff in hospitals during the COVID-19 outbreak.

**Method:**

Medical staff members in China were recruited, including frontline medical workers. The questionnaire, administered through the WeChat program, obtained demographic data and asked self-design questions related to the COVID-19 outbreak, insomnia/depressive/anxiety symptoms, and stress-related symptoms. We used a logistic regression analysis to examine the associations between sociodemographic factors and insomnia symptoms.

**Result:**

There were a total of 1,563 participants in our study. Five-hundred-and-sixty-four (36.1%) participants had insomnia symptoms according to the Insomnia Severity Index (ISI) (total score ≥ 8). A multiple binary logistic regression model revealed that insomnia symptoms were associated with an education level of high school or below (OR = 2.69, *p* = 0.042, 95% CI = 1.0–7.0), being a doctor (OR = 0.44, *p* = 0.007, 95% CI = 0.2–0.8), currently working in an isolation unit (OR = 1.71, *p* = 0.038, 95% CI = 1.0–2.8), is worried about being infected (OR = 2.30, p < 0.001, 95% CI = 1.6–3.4), perceived lack of helpfulness in terms of psychological support from news or social media with regard to COVID-19 (OR = 2.10, *p* = 0.001, 95% CI = 1.3–3.3), and having very strong uncertainty regarding effective disease control (OR = 3.30, *p* = 0.013, 95% CI = 1.3–8.5).

**Conclusion:**

Our study found that more than one-third of the medical staff suffered insomnia symptoms during the COVID-19 outbreak. The related factors included education level, an isolation environment, psychological worries about the COVID-19 outbreak, and being a doctor. Interventions for insomnia among medical staff are needed considering the various sociopsychological factors at play in this situation.

## Introduction

The 2019 new coronavirus disease (COVID-19), which appeared in Wuhan, Hubei Province, China at the end of 2019, has caused widespread concern (1). The Chinese health department immediately conducted an investigation to prevent and control the spread of the disease and deployed more than 30,000 medical staff from other provinces to the frontlines to fight against the COVID-19 outbreak in Hubei. (1). As of 10 February 2020, cases of COVID-19 were no longer limited to the city of Wuhan and were being reported in 25 countries and regions including Thailand, Japan, Korea, the USA, the UK, Germany, France, Italy, Vietnam, and Singapore (1).

The outbreak of COVID-19 not only caused great public concern, but also brought about huge psychological distress, especially for medical staff. Hospital medical staff needed to be under great pressure to participate in the incident. They worried about their health and the health of their families. Furthermore, worries about contagion, the safety of colleagues and colleagues in the healthcare field tormented medical staff. They faced loneliness and rigid expectations, which can lead to anger, anxiety, insomnia, and stress related to the uncertainty of the outbreak (2). The media report on the epidemic every day, especially regarding the mortality rate of frontline medical staff, which may raise the personal danger awareness of medical staff and increase the worry felt by their families. In order to avoid nosocomial infections, the interaction of medical staff with colleagues and patients was discouraged, leading to a further increase in isolation. As the knowledge regarding COVID-19 deepens, the infection control program needs to be continuously updated and modified. Medical staff spend hours each day putting on and removing airtight protective equipment, an increased workload caused by the COVID-19 outbreak, making them feel exhausted. After work, medical staff are easily crowded out by people around them, such as their neighbors, because the general population easily misunderstand that medical staff are especially susceptible to carrying the virus when returning home. Medical staff may also be worried about becoming infected or infecting their family members due to their own potential improper handling of the process at work. In other words, medical staff become stressed once an outbreak occurs (2).

Stress is considered the primary cause of insomnia (3, 4). Although much research has been published identifying insomnia and related psychological effects of working in hospitals during the previous SARS outbreak (5), to date there has been no study on the various risk factors that may make medical staff more susceptible to insomnia during the COVID-19 outbreak. In our study, we aimed to investigate the prevalence of insomnia symptoms and to confirm the related social psychological factors among medical staff in hospitals during the COVID-19 outbreak.

## Materials and Methods

### Study Design

We conducted a survey using a self-administered questionnaire delivered through the internet. Data were collected in China from 29 January to 3 February 2020.

### Sample and Procedure

We recruited hospital staff from all over China, including the frontline medical workers in Wuhan. Participants who met the following criteria were included: (1) hospital staff, (2) could read a Chinese questionnaire, (3) WeChat users, (4) volunteered for the survey, and (5) could submit survey responses using the same IP address only once. Exclusion criteria included: being unable to understand the questionnaire. Our investigators forwarded the questionnaire to different WeChat groups of medical staff to recruit participants. Our questionnaire was set to proceed only when each option was completed before the final submission. People who completed the questionnaire were also encouraged to forward the survey to others. We obtained a total sample of 1,946 participants. Prior to data analysis, we excluded 383 (19.7%) of the initial respondents they were identified as non-medical staff, leaving a total of 1,563 questionnaires to analyze. The study was approved by the Ethics Committee of Nanfang Hospital of Southern Medical University. All subjects provided informed consent to participate in the study. To protect the respondents' privacy, the survey was conducted anonymously.

### Measurement Tools

Using the questionnaire, we collected demographic data, asked self-design questions related to the COVID-19 outbreak, and administered the insomnia scale, the depressive/anxiety symptom scale, and the impact of event scale.

The Insomnia Severity Index (ISI) was used to measure the severity of insomnia. Each item is rated on a 0–4 scale, and the total score ranges from 0 to 28. A higher score suggests more severe insomnia symptoms. A total score of ≥8 is considered as having symptoms of insomnia (6, 7).

The Patient Health Questionnaire 9-item depression module (PHQ-9) is used to measure depressive symptoms. Each of the nine items is scored on a scale from 0 to 3. The total score suggests different levels of depressive symptoms: minimal/no depression (0–4), mild (5–9), moderate (10–14), or severe (15–21) (8, 9).

The Generalized Anxiety Disorder (GAD) scale is a recently developed simple 7-item tool based on DSM-IV criteria that can easily screen anxiety symptoms. The total score can be categorized into four severity groups: minimal/no anxiety (0–4), mild (5–9), moderate (10–14), or severe (15–21) (10).

The Impact of Events Scale-Revised (IES-R) was used to evaluate the psychological response associated with trauma. The total score can be categorized into four clinical levels according to the degree of symptoms and reactions: subclinical range (0–8), mild range (9–25), moderate range (26–43), and severe range (44–88) (11).

### Statistical Analysis

The statistical analyses were performed using IBM SPSS Statistics for Windows (version 21.0., IBM Corp., Armonk, NY, USA). Descriptive statistical analyses (*n*, %) were performed using the chi-square test. Post hoc analyses were performed on multiple category data with a Bonferroni correction. To examine the associations between demographic factors and insomnia, multiple binary logistic regression was used. Significant differences were all included in the regression model except for PHQ-9, GAD-7 and IES-R scores because of high collinearity (12). A *p*-value of <0.05 was considered statistically significant.

## Results

**Tables 1A**, **B** shows the characteristics of, and differences between the insomnia and non-insomnia groups in terms of demographic data, living situation in the past week, COVID-19 outbreak-related questions, and mood status. There were 1,563 participants in our study. Five-hundred-and-sixty-four (36.1%) participants had symptoms of insomnia according to the ISI score. The prevalence of depressive, anxiety, and stress-related symptoms was 50.7% (PHQ-9 ≥5), 44.7% (GAD-7≥5), and 73.4% (IES-R ≥9), respectively, among medical staff. All participants were divided into two groups, the insomnia group (total score ≥ 8) and the non-insomnia group (total score < 8). Individuals reporting symptoms of insomnia were more likely to be female, more likely to live in the Hubei province, less likely to live in the city, more likely to be in contact with feverish or infected patients, more likely to have been infected or live with people who have been infected, less likely to have received sufficient infection prevention training for COVID-19, less likely to use strict self-protection to avoid getting infected, less likely to think current protection can avoid getting infected, and more worried about being infected compared with those in the non-insomnia group (**Figure 1**). There were no significant differences in marital status (*p* = 0.053), workplace (*p* = 0.358), or living in the city (*p* = 0.067) between the insomnia and non-insomnia groups.

**Table 1A:** Comparisons between insomnia and non-insomnia individuals used chi-square tests on demographic data and living situations.

	Insomnia (*n* = 564)	Non-insomnia (*n* = 999)	*p*
**Demographic data**			
Gender (female)	488 (86.5)	805 (80.6)	0.003
Age (years old)			
18–25 *	121 (21.5)	160 (16.0)	0.031
26–30	154 (27.3)	291 (29.1)	
31–40	177 (31.4)	318 (31.8)	
41–50	86 (15.2)	155 (15.5)	
51–60	25 (4.4)	66 (6.6)	
60 above	1 (0.2)	9 (0.9)	
Marital status			
Single	201 (35.6)	326 (32.6)	0.053
Married	344 (61.0)	655 (65.6)	
Widow or divorced	19 (3.4)	18 (1.8)	
Education level			
High school or below *	18 (3.2)	15 (1.5)	0.002
Bachelor's degree *	450 (79.8)	747 (74.8)	
Master's degree	63 (11.2)	139 (13.9)	
Doctoral degree *	33 (5.9)	98 (9.8)	
Staff type			
Doctor *	124 (22.0)	330 (33.0)	< 0.001
Nurse *	395 (70.0)	589 (59.0)	
Hospital administration	11 (2.0)	19 (1.9)	
Other medical staff	34 (6.0)	61 (6.1)	
Staff title			
None	79 (14.0)	113 (11.3)	0.006
Junior	263 (46.6)	445 (44.5)	
Middle	166 (29.4)	281 (28.1)	
Sub-senior *	46 (8.2)	114 (11.4)	
Senior *	10 (1.8)	46 (4.6)	
Working place			
Grade III hospital	475 (84.2)	863 (86.4)	0.358
Grade II hospital	73 (12.9)	117 (11.7)	
Grade I hospital or community service or disease control center or other	16 (2.8)	19 (1.9)	
Current working department			
Fever outpatient	23 (4.1)	51 (5.1)	< 0.001
Emergency *	33 (5.9)	27 (2.7)	
Isolation unit *	127 (22.5)	129 (12.9)	
Intensive care unit	51 (9.0)	70 (7.0)	
Normal outpatient or impatient unit*	275 (48.8)	608 (60.9)	
Other (administration or logistics staff)	55 (9.8)	114 (11.4)	
**Living situation in past week**			
Living in Hubei province (yes)	432 (76.6)	686 (68.7)	0.001
Living in the city or country (city)	539 (95.6)	972 (97.3)	0.067
Living arrangement			
Alone	153 (27.1)	229 (22.9)	< 0.001
With family *	284 (50.4)	609 (61.0)	
With friend or colleague *	119 (21.1)	152 (15.2)	
With others	8 (1.4)	9 (0.9)	

*Significant after Bonferroni correction.

**Table 1B:** Comparisons between insomnia and non-insomnia individuals used chi-square tests on COVID-19 outbreak-related questions, and mood status.

	Insomnia (*n* = 564)	Non-insomnia (*n* = 999)	*p*
**COVID-19 outbreak-related questions**			
Work requires contact with feverish or infected patients (yes)	302 (53.5)	387 (38.7)	<0.001
You or people living with you got infected (yes)	120 (21.3)	144 (14.4)	0.001
Got sufficient infection prevention training for COVID-19 (yes)	388 (68.8)	754 (75.5)	0.004
Strict self-protection for COVID-19 (yes)	435 (77.1)	841 (84.2)	0.001
Current protection can prevent getting infected (yes)	361 (64.0)	768 (76.9)	<0.001
Worried about being infected (yes)	520 (92.2)	776 (77.7)	<0.001
Psychological support from news or social media regarding COVID-19			
Very helpful *	106 (18.8)	271 (27.1)	<0.001
Somewhat helpful	117 (20.7)	195 (19.5)	
A little helpful	140 (24.8)	220 (22.0)	
Not helpful *	67 (11.9)	59 (5.9)	
No comment	134 (23.8)	254 (25.4)	
Hours each day spent on reading information about the COVID-19 outbreak in past week			
< 1 hour/day	78 (13.8)	173 (17.3)	0.012
1–2 hours/day	225 (39.9)	437 (43.7)	
3–4 hours/day	138 (24.5)	229 (22.9)	
≥5 hours/day *	123 (21.8)	160 (16.0)	
Uncertainty regarding effective disease control			
Very strong *	157 (27.8)	134 (13.4)	<0.001
Strong *	228 (40.4)	348 (34.8)	
Low *	173 (30.7)	481 (48.1)	
None *	6 (1.1)	36 (3.6)	
Impact of event			
Sub-clinical *	19 (3.4)	397 (39.7)	<0.001
Mild *	135 (23.9)	427 (42.7)	
Moderate *	241 (42.7)	158 (15.8)	
Severe *	169 (30.0)	17 (1.7)	
**Mood status**			
Depressive symptoms			
Minimal/no *	82 (14.5)	689 (69.0)	<0.001
Mild *	259 (45.9)	264 (26.4)	
Moderate *	129 (22.9)	28 (2.8)	
Severe *	94 (16.7)	18 (1.8)	
Anxiety symptoms			
Minimal/no *	109 (19.3)	755 (75.6)	<0.001
Mild *	286 (50.7)	211 (21.1)	
Moderate *	103 (18.3)	16 (1.6)	
Severe *	66 (11.7)	17 (1.7)	

*Significant after Bonferroni correction.

**Figure 1:**
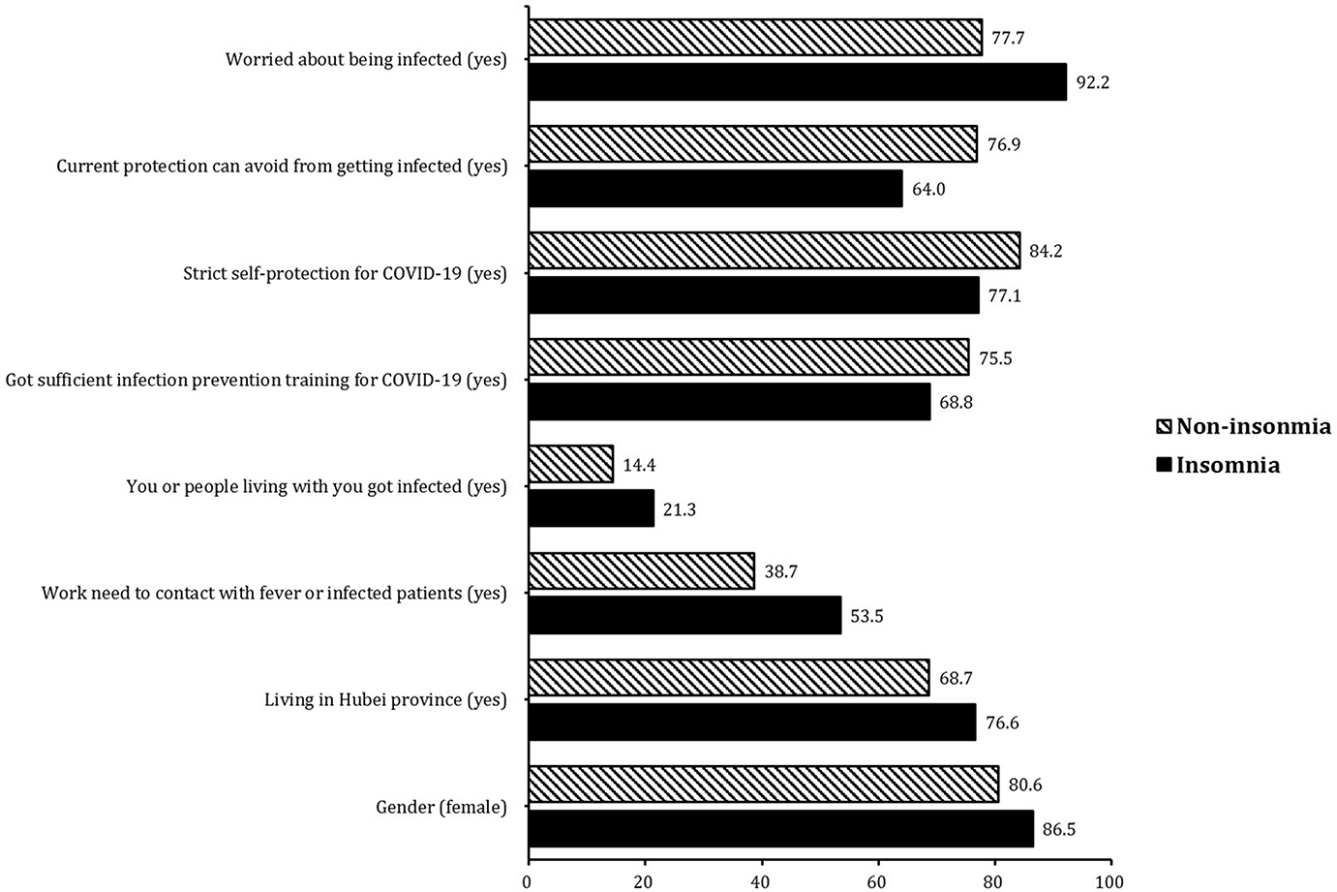
Significant differences between the insomnia group and non-insomnia group in demographic data and sociopsychological factors.

**Figure 2:**
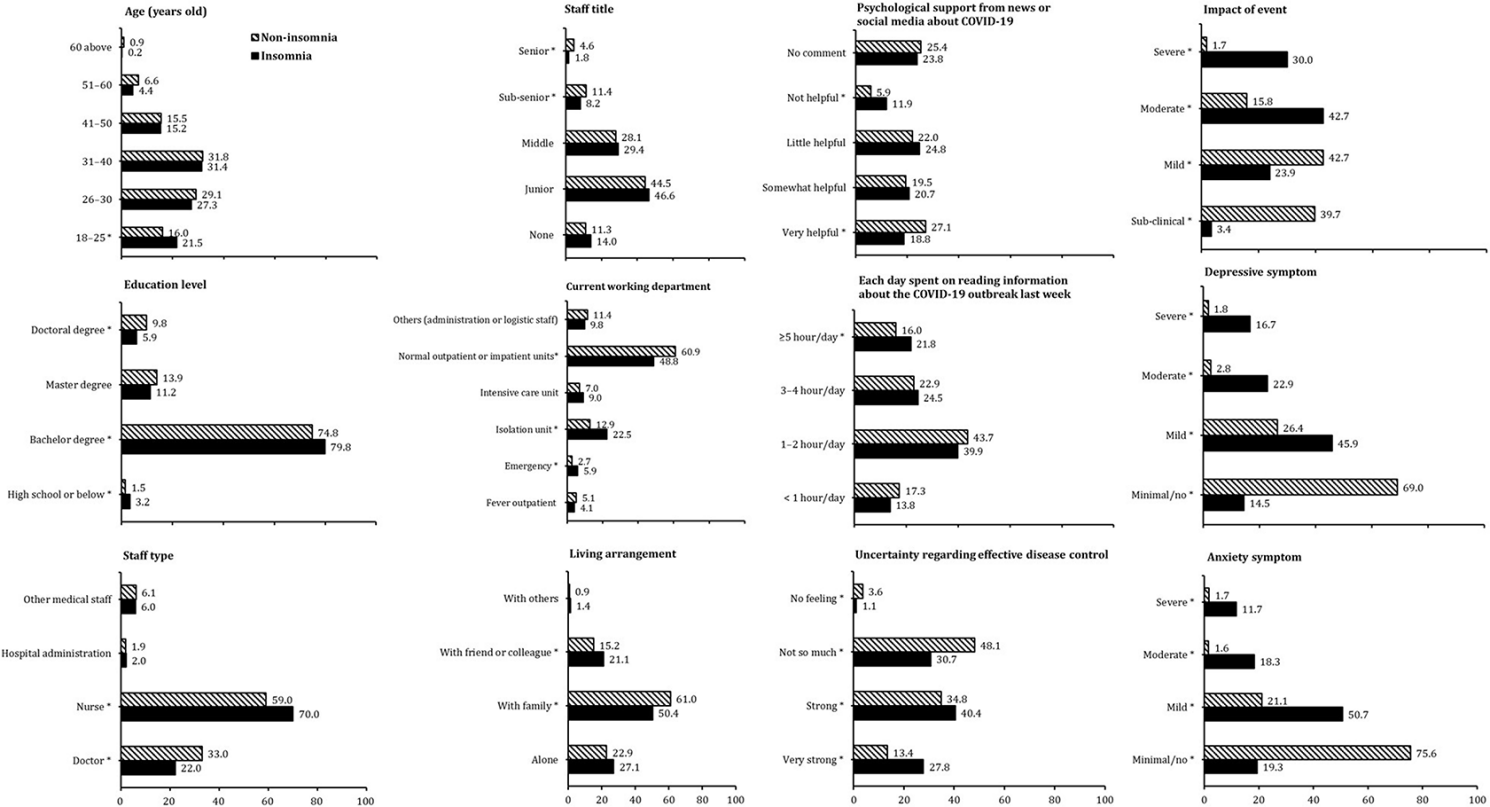
Post hoc analyses of multiple comparisons between the insomnia and non-insomnia group on demographic data, sociopsychological factors, and mood status, *Significance after Bonferroni correction.

**Figure 2** shows the multiple comparison results of *post hoc* analyses with Bonferroni correction. Individuals of the insomnia group were more likely to be in the 18–25 age range (21.5% vs 16.0%), have a high school education or below (3.2% vs 1.5%) and a bachelor's degree (79.8% vs 74.8%), be nurses (70.0% vs 59.0%), work in an emergency room (5.9% vs 2.7%) and isolation unit (22.5% vs 12.9%), live with friends or colleagues (21.1% vs 15.2%), think that the psychological support from the news and social media is not helpful (11.9% vs 5.9%), spent ≥ 5 h reading information about the COVID-19 outbreak in the last week (21.8% vs 16.0%), feel very strong uncertainty regarding effective disease control (27.8% vs 13.4%) and strong uncertainty regarding effective disease control (40.4% vs 34.8%), think that the impact of the event is moderate to severe (42.7% vs 15.8%; 30.0% vs1.7%), have significantly higher scores of depressive and anxiety symptoms on mild (45.9% vs 26.4% and 50.7% vs 21.1%), moderate (22.9% vs 2.8% and 18.3% vs 1.6%), and severe (16.7% vs 1.8% and 11.7% vs 1.7%) levels when compared with those in the non-insomnia group. The insomnia group was smaller than the non-insomnia group and included participants with a doctoral degree (5.9% vs 9.8%), doctors (22.0% vs 33.0%), with sub-senior (8.2% vs 11.4%) and senior (1.8% vs 4.6%) titles, that live with family (50.4% vs 61.0%), that think that the psychological support from the news and social media it very helpful (18.8% vs 27.1%), feel not so much (30.7% vs 48.1%) and have no feeling (3.6% vs 1.1%) on the uncertainty regarding effective disease control, think that the impact of the event is sub-clinical (39.7% vs 42.7%) or mild (42.7% vs 23.9%), and have minimal/no depressive and anxiety symptoms (14.5% vs 69.0% and 19.3% vs 75.6%) (**Tables 1A**, **B**).

**Table 2** shows the multiple binary logistic regression results of insomnia. Significant factors were found on education level (high school or below) (OR = 2.685, *p* = 0.042), staff type (doctor) (OR = 0.437, *p* = 0.007), current working department (isolation unit) (OR = 1.708, *p* = 0.038), worry about being infected (OR = 2.299, *p* < 0.001), perceived psychological support from news or social media regarding COVID-19 (not helpful) (OR = 2.095, *p* = 0.001), and uncertainty regarding effective disease control (very strong) (OR = 3.297, *p* = 0.013).

**Table 2:** Multiple binary logistic regression analysis of insomnia-related factors.

Variable	B	SE	OR	*p*	95% CI
**Demographic data**					
Gender (female)	0.221	0.188	1.247	0.240	0.863–1.802
Age (years old) (ref: 18–25)					
26–30	−0.294	0.178	0.745	0.099	0.526–1.057
31–40	−0.205	0.207	0.814	0.322	0.542–1.223
41–50	0.064	0.266	1.066	0.809	0.633–1.797
51–60	0.108	0.366	1.114	0.768	0.543–2.284
60 or above	−1.362	1.158	0.256	0.240	0.026–2.480
Education level (ref: doctoral degree)					
High school or below	0.988	0.487	2.685	0.042	1.034–6.971
Bachelor's degree	0.077	0.281	1.080	0.785	0.622–1.873
Master's degree	0.138	0.281	1.148	0.623	0.662–1.989
Staff type (ref: other medical staff)					
Doctor	−0.828	0.307	0.437	0.007	0.240–0.797
Nurse	−0.438	0.287	0.645	0.126	0.368–1.131
Hospital administration	−0.341	0.491	0.711	0.487	0.271–1.862
Staff title (ref: senior)					
None	0.322	0.495	1.381	0.515	0.523–3.641
Junior	0.267	0.468	1.306	0.568	0.522–3.266
Middle	0.411	0.434	1.509	0.343	0.644–3.534
Sub-senior	0.193	0.429	1.212	0.654	0.523–2.813
Current working department (ref: other [administration or logistics staff])					
Fever outpatient	−0.043	0.346	0.958	0.900	0.486–1.885
Emergency	0.599	0.355	1.820	0.092	0.907–3.651
Isolation unit	0.535	0.258	1.708	0.038	1.030–2.833
Intensive care unit	0.178	0.287	1.195	0.534	0.681–2.097
Normal outpatient or impatient units	−0.092	0.214	0.912	0.666	0.599–1.387
**Living situations in last week**					
Living in Hubei province (yes)	0.087	0.152	1.091	0.566	0.810–1.469
Living arrangement (ref: with others)					
Alone	0.096	0.559	1.101	0.864	0.368–3.290
With family	−0.139	0.552	0.870	0.802	0.295–2.568
With friend or colleague	−0.016	0.562	0.984	0.978	0.327–2.960
**COVID-19 outbreak-related questions**					
Work requires contact with feverish or infected patients (yes)	0.224	0.136	1.252	0.098	0.960–1.632
You or people living with you got infected (yes)	0.183	0.151	1.201	0.227	0.893–1.615
Got sufficient infection prevention training for COVID-19 (yes)	0.029	0.140	1.029	0.838	0.782–1.354
Strict self-protection for COVID-19 (yes)	−0.235	0.162	0.791	0.148	0.575–1.087
Current protection can prevent getting infected (yes)	−0.240	0.141	0.787	0.089	0.597–1.037
Worried about being infected (yes)	0.833	0.194	2.299	<0.001	1.573–3.360
Psychological support from news or social media regarding COVID-19 (ref: no comment)					
Very helpful	−0.152	0.172	0.859	0.377	0.614–1.203
Somewhat helpful	0.263	0.172	1.301	0.126	0.929–1.823
A little helpful	0.228	0.164	1.256	0.165	0.910–1.734
Not helpful	0.740	0.231	2.095	0.001	1.333–3.295
Hours each day spent on reading information about the COVID-19 outbreak in past week (ref: < 1 hour/day)					
1–2 hour/day	0.195	0.172	1.216	0.256	0.868–1.703
3–4 hours/day	0.270	0.189	1.310	0.153	0.905–1.898
≥5 hours/day	0.334	0.199	1.396	0.094	0.945–2.063
Uncertainty regarding effective disease control (ref: no feeling)					
Very strong	1.193	0.481	3.297	0.013	1.284–8.469
Strong	0.745	0.472	2.107	0.114	0.835–5.317
Low	0.204	0.471	1.227	0.665	0.487–3.090

## Discussion

As far as we know, this is the first study concerning insomnia symptoms among medical staff during the COVID-19 outbreak. Our study found that 36.1% of medical staff suffered from insomnia among the 1,563 participants. The insomnia group had more psychological problems related to the COVID-19 outbreak. We found that low educational level, an environment of isolation, worry about being infected by COVID-19, perceived unhelpfulness of psychological support from the news or social media regarding the COVID-19 outbreak, and extreme uncertainty regarding effective disease control of the COVID-19 outbreak were all risk factors for insomnia, while the staff type of doctor was a protective factor.

The prevalence rate of insomnia was 36.1% among medical staff in our study, which was consistent with 34.2% in Hong Kong and 37% in Taiwan during the SARS epidemic (13, 14). Stress involves increased psychological and physical activation in response to demand, and the activated hypothalamus-pituitary-adrenal (HPA) system is incompatible with normal sleep (15). The resulting sleep disorders may appear to lead to further increases in the HPA system, thereby promoting a vicious cycle of stress and insomnia (15). A longitudinal study showed that during the SARS outbreak, the sleep quality among medical staff was worst during the crisis and gradually improved after 2 weeks, suggesting that insomnia was related to contagion outbreak-induced stress. Obviously, the main source of stress among medical staff in the present study was from the COVID-19 epidemic.

We found that the risk of insomnia among medical staff with an education level of high school or below was 2.69-times higher than that of those with a doctoral degree. This was consistent with an insomnia survey of the general population in China that found that a low education level was associated with a high possibility of insomnia (16). However, another study, during the SARS epidemic, found that sleep quality was not affected by the education level of nurses (17). In Chen's study (17), the nurses were mostly from junior colleges (68.1%), and the lowest education level was a vocational school. Our study included 31 individuals with high school level education and two individuals with middle school education level or below, indicating poor comprehension ability. Individuals with a low educational level may have difficulty understanding the outbreak compared with those with a doctoral degree. Another study of the SARS epidemic in China showed that a low education level was related to the fear of SARS (18). Thus, the fear of the COVID-19 outbreak may particularly impact the sleep quality of individuals with a low education level.

Furthermore, medical staff working in an isolation environment had a 1.71-times higher probability of experiencing insomnia. Isolation has been the main mode of treatment during the COVID-19 outbreak. A study on a SARS isolation unit reported that patients were fearful, lonely, bored, and angry (19). The medical staff were also worried about the effect of quarantine and contagion on family members, friends, and colleagues (20). Our study also showed depressive and anxiety symptoms among medical staff during the COVID-19 outbreak. Previous research has found that the length of time in isolation predicted anger and avoidance behaviors, and those in longer isolation would find more negative outcomes (21). Moreover, the medical staff are in a situation where they are helping and caring for others while being exposed to the contagion itself. After brief training, medical staff were incorporated into the frontline battle against COVID-19. Additionally, it was not possible to set up isolation rooms consisting of an anteroom and clean zone because of insufficient equipment once the hospital rapidly became a designated COVID-19 center. Medical staff must be equipped with full-body protective equipment under negative pressure for more than 12 h, including double-layer protective equipment, double-face masks, double-layer gloves, isolation caps, foot covers, and protective glasses. To avoid being infected while removing protective equipment, staff members cannot eat, drink, or use the bathroom during working hours. Many of them are dehydrated due to excessive sweating, and some develop cystitis and a rash. Medical staff working in the quarantine area must always maintain close contact with infected cases. Under these dangerous conditions, medical staff become mentally and physically exhausted, and therefore experience an increased risk of insomnia due to high stress.

Factors related to insomnia during the COVID-19 outbreak included worry about being infected by COVID-19, perceived unhelpfulness of psychological support form news or social media regarding the COVID-19 outbreak, and extreme uncertainty regarding effective disease control of the COVID-19 outbreak. We also found that the impact of the event was more severe in the insomnia group than in the non-insomnia group, suggesting a significant impact of the COVID-19 epidemic on sleep quality. In a nurse's study, higher trust in infection control devices and procedures predicted less emotional fatigue and anger (21). Previous research has also found that greater concerns about the risk of infection were related to medical staff's concerns about personal or family health (22). It is believed that a higher level of trust in workplace precautions is related to a decline in the focus on the outbreak, which was also in line with our study (23). The worries among medical staff may impact sleep quality induced by anxiety symptoms (24). During and in the aftermath of an outbreak, about one in six medical staff members developed significant stress symptoms (25). In most surveys, prior preparation was important in all respects, suggesting that there were clear plans, policies and procedures, and occasional exercises can have a significant psychological impact. Understanding what is happening, responding to it, how medical staff adapt to the overall operation and their own roles and expectations can help medical staff focus on key issues avoiding the uncertainty that creates anxiety. Frequent policy changes, unclear standards for case management, and other ambiguities during the crisis have led to depression, stress, and anxiety (26). In addition to stress, medical staff may suffer from insomnia symptoms due to circadian disturbance. For the majority of shift workers this circadian misalignment is temporary with recovery within a few days after returning to a normal sleep wake schedule. However, during the outbreak, medical staff were working around the clock to save people's lives. The increased workload substantially impairs medical staff's ability to sleep, resulting in insomnia, severe sleep debt and daytime sleepiness.(27).

Unexpectedly, we found that the staff type of doctor seemed to be a protective factor for insomnia. Consistent with this, a study from Singapore after SARS found that doctors scored lower than nurses on posttraumatic stress (28). Another study also found greater levels of stress in nurses than in doctors, which indicated that nurses were also more likely to have an increased workload (29, 30). Doctors often work in the daytime, so that they can get good sleep at night, while nurses may have to work the whole night with frequent night shifts (27). Nurses are more likely to have circadian rhythm dysfunctions induced by irregular and frequent night shifts (31). Additionally, doctors tend to have a higher level of education. In our study, only one doctor had a high school or below education level, while 129 doctors had a doctoral degree. Furthermore, a previous study showed that more contact with patients with higher-severity illness resulted in higher IES scores (32). Doctors often have less contact with patients than nurses. Finally, most nurses in our study were female, while the doctors were mostly male. According to a meta-analysis, females are more susceptible to insomnia (33).

Our findings can help provide precise interventions of insomnia for medical staff, especially for those with different sociopsychological risk factors. Cognitive insomnia behavior therapy (CBTI) can effectively treat acute insomnia. It aims to address the cognitive and behavioral factors of permanent insomnia, including a range of therapeutic components such as sleep hygiene education, relaxation therapy, stimulation control, sleep restriction, and cognitive therapy (34). In addition, CBTI may improve patients' self-efficacy and confidence in controlling their sleep problems and is recommended as a first-line treatment for acute insomnia in adults. Treatment guidelines should be updated to reflect our findings. The risk factors, such as low education level, being in an isolation unit, and staff type could help psychiatrists screen the susceptible population rapidly and offer personalized treatment. Understanding the problems found in the insomnia group can help hospital administrations with effective mental health education and training among medical staff.

This was the first study to focus on the sleep quality of medical staff during the COVID-19 outbreak. Our study has several limitations. First, the survey was a self-report questionnaire based on the WeChat program. We conducted a rapid survey due to the time limitation of the epidemic. Second, only the ISI was used to assess insomnia symptoms. The score cutoff of 8 is widely accepted to detect insomnia symptoms. Importantly, the medical staff were all busy with the battle of the COVID-19 outbreak; complicated tools may burden them with a higher requirement of attention. Third, a self-designed questionnaire was used. There is no standard questionnaire for the investigation of sociopsychological factors during a contagion outbreak.

## Conclusion

Our study found that more than one-third of the medical staff suffered from insomnia symptoms during the COVID-19 outbreak. The related factors included education level, isolation environment, worries about the COVID-19 outbreak, and occupation. Interventions for insomnia are needed to target different sociopsychological factors.

## Data Availability Statement

The dataset compiled for this study is available upon reasonable request to the corresponding authors.

## Ethics Statement

The studies involving human participants were reviewed and approved by the Institutional Review Board, Nanfang Hospital of Southern Medical University. All subjects provided informed consent to participate in the study. To protect the respondents' privacy, the survey was conducted anonymously.

## Author Contributions

BZ and ZL conceived and designed this study, and CZ made additional contributions to its design. CZ conceived and conducted statistical analyses, with additional advice regarding analyses contributed by LY, SL, SM, YW, ZC, HD, RL, LK, MS, and JZ. CZ drafted the manuscript, and all authors contributed to editing it and approved the final manuscript.

## Funding

This study was funded by the President Foundation of Nanfang Hospital, Southern Medical University (2019Z014), the Key Item of Guangzhou Bureau of Education (2019KC106), the National Natural Science Foundation (81901348), and the National Key R&D Program of China (2018YFC1314600).

## Conflict of Interest

The authors declare that the research was conducted in the absence of any commercial or financial relationships that could be construed as a potential conflict of interest.
